# Assessing and Validating the Ability of Machine Learning to Handle Unrefined Particle Air Pollution Mobile Monitoring Data Randomly, Spatially, and Spatiotemporally

**DOI:** 10.3390/ijerph191610098

**Published:** 2022-08-16

**Authors:** Asmaa Alazmi, Hesham Rakha

**Affiliations:** 1 Department of Construction Project, Ministry of Public Work of Kuwait, Kuwait City 12011, Kuwait; 2 Department of Civil and Environmental Engineering, Virginia Polytechnic Institute and State University, Blacksburg, VA 24061, USA

**Keywords:** machine learning, land use regression, black carbon, particulate number, spatial and temporal variation, air pollution

## Abstract

Many epidemiological studies have evaluated the accuracy of machine learning models in predicting levels of particulate number (PN) and black carbon (BC) pollutant concentrations. However, few studies have investigated the ability of machine learning to predict the pollutant concentration with using unrefined mobile measurement data and explore the reliability of the prediction models. Additionally, researchers are moving away from using fixed-site data in favor of using mobile monitoring data in a variety of locations to develop hourly empirical models of particulate air pollution. This study compared the differences between long-term (daily average) and short-term (hourly average and 1 s unrefined data) model performance in three different classes of cross validation: randomly, spatially, and spatially temporally. This study used secondary data describing BC and PN pollutant levels in the rural location of Blacksburg (VA). Our results show that the model based on unrefined data was able to detect the pollutant hot spot areas with similar accuracy compared to the aggregated model. Moreover, the performance was found to improve when temporal data added to the model: the 10-fold MAE for the BC and PN were 0.44 μg/m^3^ and 3391 pt/cm^3^, respectively, for the unrefined data (one second data) model. The findings detailed here will add to the literature on the correlation between data (pre)processing and the efficacy of machine learning models in predicting pollution levels while also enhancing our understanding of more reliable validation strategies.

## 1. Introduction

Estimating the impacts of long-term exposure to ambient air pollution is crucial for determining a range of possible health-related risks, such as cardiovascular and respiratory diseases. The World Health Organization (WHO) published a report in 2018 indicating that outdoor air pollution resulted in approximately 4.2 million premature deaths worldwide. BC (black carbon) and PN (particulate number) are thought to act as common carriers of various chemicals that are harmful to the human body; when inhaled, BC and PN can penetrate the circulatory system and cause complex biological responses [1,2]. For instance, BC and PN have been linked to adverse neonatal risks, including low birth weight and preterm birth [3,4], and, of course, the presence of these pollutants in the atmosphere are known contributors to global warming [5,6]. Furthermore, particulate matter is associated with numerous health issues and negative health outcomes, including respiratory and cardiovascular disorders [7], lung cancer [8], asthma [9], and an overall shorter lifespan [10].

Intra-urban air pollution is distinguished by the high geographical diversity of contaminants, as well as rapid destruction from the source [11]. Sulfur concentrations, for example, have been shown to decrease by 50% between 50 and 150 m from a highway [12]. Nitrogen dioxide levels have been shown to be approximately 2.5 times higher within 50 m of the source compared to beyond that range [13]. Increasingly, the concentration of air pollution in an area has been investigated using mobile air quality monitoring tools (instead of focusing on a fixed site), which allow for more accurate and comprehensive assessments of air quality [12]. A recent study compared prediction estimates from mobile and short-term stationary land-use regression models (LUR) [14]. Land-use regression modeling (LUR) has been used globally to classify air pollution exposure, predict the presence and level of traffic-related pollutants, and identify the potential health impacts on people living in urban areas. This study utilized a number of land-use variables (e.g., road type, traffic count, elevation, and vehicle emissions) as input variables in regression analysis to build an empirical predictive model of pollutant concentration. Land-use regression is a popular method widely used for analyzing, explaining, and predicting air pollution concentrations—particularly in areas that are densely populated. According to Morley and Gulliver (2018), the model relies on predictable air-pollution patterns to help estimate pollution patterns within a particular area [15]. Regression equations are then used to describe the relationship between environmental variables and the target locations. Using both mobile and stationary monitoring data, the researchers confirmed the strong correlation between ultrafine particle and black carbon concentration predicted by LUR models. However, the researchers also reported that predicted concentrations based on mobile measurements were consistently higher.

The use of land-use regression (LUR) models represents a widely used approach for assessing urban air pollution. However, various machine learning (ML) modeling strategies have recently been developed to overcome the shortcomings of LUR in capturing non-linear interactions between contaminants and predictors. In general, ML models outperform LUR models when used to assess the same contaminants [16]. A recent study designed to valuate different ML algorithms to predict ${\mathrm{PM}}_{2.5}$ concentrations demonstrated that the ML approaches were clearly superior to LUR modeling [17]. Researchers also compared linear regression and a machine learning approach for ambient ultrafine particles, estimating that standard multivariable regression represented 62% of the spatial variation of ultrafine particles while the ML algorithm explained 79% of the variance [18]. Another study used stationary and non-stationary ultrafine particle data obtained in the Netherlands to evaluate linear and non-linear regression; the authors demonstrated that performance deteriorated when external spatial validation was applied [19].

Machine learning algorithms designed for use in spatiotemporal data applications, which can be used to forecast a specific concentration for unknown locations, enable ambient air pollution monitoring. However, the specific validation strategy is important for assessing a given model’s performance to avoid model overestimation. One study indicated that the coefficient of determination R^2^ decreased from 0.82 to 0.75 when leave-one-out cross-validation was applied [20]. Another drawback is that the majority of LUR models are designed for specific cities, limiting their application to other regions [21]. Validation strategies have been applied differently in spatiotemporal modeling. For example, researchers have applied both spatial validation and temporal validation to define the ML model uncertainty of a nitrogen dioxide (NO_2_) prediction model [22]. Recently, researchers applied both cross-validation and external validation to define the performance of annual average fine particle matter and nitrogen dioxide concentration prediction models [23]. The coefficient of determination (R^2^) was found to decrease from ~0.63 to ~0.59 for fine particle concentration, and from ~0.59 to 0.50 for nitrogen dioxide. Earlier researchers applied ML toward developing prediction models that have spatial and temporal structures that introduce spatiotemporal autocorrelation [24].

Estimating the short-term exposure risk to ambient air pollution continues to be a challenging epidemiological task due to significant small-scale spatial heterogeneity. As such, effective methods for capturing spatiotemporal variations in the distribution of pollutants are becoming increasingly important. Accordingly, our investigation was designed to add to the growing body of literature in this area by assessing BC and PN concentration model uncertainty with high spatiotemporal resolution.

## 2. Materials and Methods

For this study, measurements of particulate air pollution were collected via mobile monitoring within a small rural college town (Blacksburg, VA; Population: 181,863) during the daylight hours (7 am to 7 pm) of the summer and fall of 2016. These data were then used to develop several ML models to test the ability of ML to handle noisy, unprocessed mobile monitoring data vs. using spatially and temporally smoothed inputs. We focused on comparing how model-derived short-term concentration estimates (hourly average: hourly aggregated data spatially 100 m and temporally 1 h, and one second: the disaggregated raw data) compared to long-term estimates (daily average model: 12 h average) with the goal of evaluating how the design of a short-term modeling approach affects exposure assessment. Additionally, we also compared the performance of these models to the models produced by stepwise regression model to enable performance comparisons of the ML models to analogous data obtained from traditional stepwise regression models. We used three types of cross-validation in the short-term models (random, spatial, and spatial–temporal cross validation) to assess the performance of the reliable model to forecast the pollutant concentration in an unseen location. All the models were built using Python 3.7 and scikit-learn library and implemented in Advanced Research Computing (ARC) at Virginia Tech.

### 2.1. Data Collection

**Figure 1:**
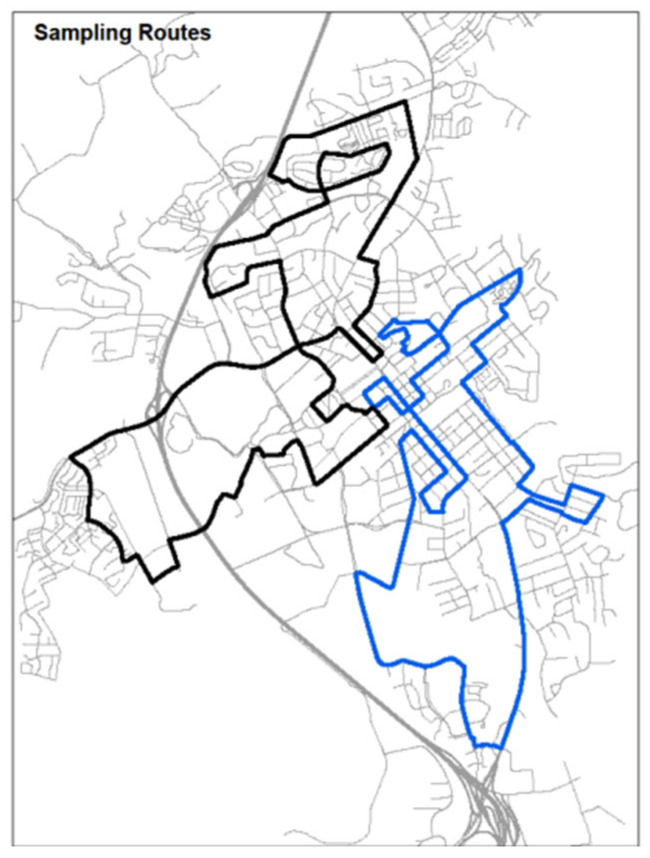
Sampling bicycle routes [28].

The bicycle-based mobile that measured particle size and fine particulate matter (${\mathrm{PM}}_{2.5}$), as well as particle number (PN) and black carbon (BC) in Blacksburg, VA, were used to develop empirical ML models. PN and BC are pollutants that are known tracers of traffic emissions [25], have a high degree of spatial variability in urban areas [26], and are linked to a range of health disparities [27]. Short-term mobile monitoring data were collected based on a prior study [28] and the details for that data collection campaign and bicycle routes can be found there. To summarize the process, however, a mobile monitoring platform was set up during the summer and fall of 2016. Microaethalometers (AE51; AethLabs, Blacksburg, VA, USA) mounted on bicycles were used to measure BC concentrations, and condensation particle counters (CPC 3007; TSI, Inc., Shoreview, MN, USA) were used to measure PN concentrations. Two different monitoring routes were chosen. The sampling paths are depicted in Figure 1. Each route was 20 km long and took 45 to 60 min to complete.

Researchers have experimented with various methods for adjusting mobile monitoring data for BC and PN background concentrations in Blacksburg [28]. For model development, we used concentrations adjusted by the multiplicative method, which attempts to best approximate long-term air pollution concentrations. In brief, the multiplicative background adjustment method computed adjustment factors according to the ratio of daily concentration to hourly concentration at a central site where background concentrations were measured. Then, the adjustment factors were multiplied to all mobile monitoring observations (based on the hour the mobile measurements were collected) to account for differences in background concentrations. This monitoring effort was undertaken with four goals in mind: (a) to test the ability of noisy, unprocessed mobile monitoring data to be used in the development of empirical ML models capable of estimating short-term concentrations; (b) to compare the performance of random, spatial, and spatial temporal CV models; and (c) to introduce a systematic validation approach that could help to improve model validation strategies.

### 2.2. Modeling Approach

Previous research indicates that using more than one ML algorithm is superior to using a single model [29]. Thus, we employed nine ML algorithms based on the size of the datasets: Decision Tree, Extra Tree, KNeighbors, Support Vector Machine, Ridge, ElasticNet, Lasso, Random Forest, and Gradient Boosting. This approach assisted in the development of three sets of ML models to compare the short- and long-term concentrations calculated from the models developed using mobile monitoring data. As noted, three models were developed: one second models that included the raw disaggregated data; and two spatially and temporally aggregated models, namely daily average and hourly average. For spatial aggregation, the mobile monitoring measurements were first spatially aggregated at 100 m intervals along the monitoring path, after which the median of concentration measurements were determined for each aggregated location. This aggregation approach was applied for each hour of the day starting at 7:00 am and ending at 7:00 pm. Machine learning has the advantage of being accurate even when data are collected in the absence of a carefully controlled experimental design or when tasked to assess complicated nonlinear interactions. The aim of one second models was to test the ability of ML to capture all patterns without the need for data preprocessing, which is typically required using conventional statistical models.

Two combinations of input variables were incorporated into the model-building process: (a) spatial component (land use, transportation, and natural environment); and (b) temporal component (meteorological parameters and hour of the day). The hour of the day was added as a dummy variable (6−7 pm represents referent data). The meteorological variables for hourly temporal resolution were obtained from the Real-Time Mesoscale Analysis database. For consistency comparison between traditional regression and machine learning algorithms, we followed previous research in tabulated spatial input variables, and we used the same temporal variables [28]. In brief, the input temporal variables were temperature, relative humidity, planetary boundary layer height, precipitation, wind speed, and hour-of-day. The independent spatial variables (land use: building impervious surface; employment density; housing density; household income; industrial area; nonbuilding impervious surface; nonresidential addresses; population density; residential addresses; retail area; transportation network: all roads, bus stops, freeway, heavy duty vehicle volume, light duty vehicle volume, major roads, on-street bike facility, off-street trail, paved parking; and natural environment: nontree vegetation, tree canopy, and water) were grouped into 15 buffer sizes (25–3000 m).

We developed the daily average model using the land use data, and for the one second and hourly average models we developed two types of models based on the input variables fed into the model: (a) land use only to be consistent with daily average model when compared; and (b) land use, weather, and hour of the day (Table 1). The set of algorithms applied to each dataset varied. For example, we did not employ either KNeighboor or SVM in the models featuring all the input variables—principally because they are very computationally expensive to train using large datasets. Moreover, we used three types of cross validation in model validation process: random, spatial, spatial–temporal CV. We applied random and spatial CV in the daily average and hourly average models for both pollutants. For the one second models, we applied the random CV and spatial CV, and the spatial temporal CV (Table 1).

#### 2.2.1. Daily Average Models

We developed models designed to pool mobile measurement data obtained throughout the day to compare strategies for estimating long-term averages. We spatially aggregated all mobile monitoring data across all hours (7 am to 7 pm) as an input to our models. The resulting models show the median concentrations throughout the day. The daily average model was developed based on land-use data only. By aggregating data across all hours of the day, the mobile measurement and data aggregation approach was intended to remove temporal variables [28].

#### 2.2.2. One Second Models

We developed the one second model using raw disaggregated measurement data. This approach facilitated the prediction of pollutants across small temporal resolution to determine the ability of ML to build an empirical model using raw data. We built two types of one second models using different combinations of input variables: (a) One_second_Lu (model based on land-use data only); and (b) One_second_Lu_W_Hr (model based on land use, weather, and hourly data). Applying various combinations of input variables was intended to help determine the efficacy of ML model performance.

#### 2.2.3. Hourly Average Models

For the hourly average models, all mobile measurement data were re-aggregated for each hour of the day for each aggregation location, after which the hour of the day was added as candidate dummy independent variable. We developed the same two types for the one second models: Hourly_average_Lu, and Hourly_average_Lu_W_Hr. This approach facilitated the comparison of the short-term models to determine the ability of ML to handle the noisy, unprocessed mobile monitoring data model (one second model) vs. spatially and temporally smoothed data models (daytime and hourly average model).

### 2.3. Modeling Implementation

All the regression models were implemented using Python and scikit-learn [30], which is an open-source ML library for the Python programming language. All hyper-parameters were tuned using the random 10-fold cross-validation method and the GridSearchCV function on the model training process. We defined the estimator/algorithm type and the sequence of hyper-parameter. We then set the negative mean absolute error as a scoring method as an input to the GridSearch function. Because some ML models require the dependent variables to be similarly scaled, we standardized the independent variables. We also log transformed all mobile measurement–concentration data to fit a normal distribution more closely for modeling as a model input. It should be noted that a small number of black carbon concentration estimates evidenced zero or negative values due to noise in the micro-aethalometer results. To overcome this issue, we add 1 μg/m^3^ to the BC concentration in the model training process; all model results were then transformed back to their original values for estimating and reporting model performance statistics (e.g., error). We then validated the models using three types of cross-validation on the test data, which is described more fully in Section 2.4. Grid-SearchCV is a function used to define the optimal hyper-parameter of the model; it can exhaustively search over specified parameter values defined by the user and automatically detect the hyper-parameter that provides an accurate prediction.

### 2.4. Model Validation

Three types of k-fold cross-validation strategies were applied according to the type of dataset: (a) randomly split up into 10 groups; (b) spatially split up into 17 spatial clusters based on several spatial sets defined by k-mean clustering using spatial variables (land use, transportation); and (c) spatially temporally split up into 17 clusters using the spatial set and the run number (detailed in Section 2.4.3). For the k-fold cross validation, the quality and reliability of the models were measured using mean absolute error (MAE) and root mean squared error (RMSE); this approach enabled comparisons across different model sets and for the different number of variables for the different cross-validation approaches. Comparisons were conducted to determine the best performance algorithms for each model.

To compare the robustness and suitability of the ML model with traditional statistically based models, the coefficient R^2^ for both daily average and hourly average random CV models was determined. Additionally, random cross validation was applied for all model types (daily average, hourly average, and one second) to assess the model overestimation. We applied three strategies of CV for the one second models (random, spatial, and spatial–temporal CV) to facilitate comparing the performance of the spatial and spatial temporal CV to the random CV using very small temporal resolution data. It should also be noted that we did not apply the spatial–temporal CV in daily average and hourly average models—mainly due to the fact than when we increased the restriction of the hold-out test data we lost observations in the test set. Thus, any test errors should not be considered to be representative.

#### 2.4.1. Random Cross-Validation

Random cross-validation is intended to determine a model’s performance at a random time and location. For this study, random CV was calculated as follows. First, air pollution measurements were randomly split into 10 groups. Then, we selected one group as the hold-out group, trained the model using the remaining available data, and then predicted air pollution concentrations for the hold-out group. This process was repeated separately for each of the 10 groups.

#### 2.4.2. Spatial Cross-Validation

**Figure 2:**
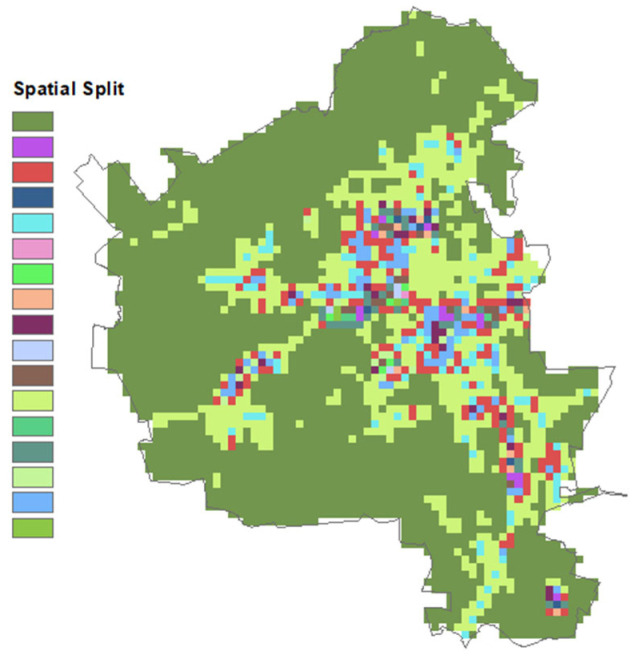
The 17 identified spatial clusters.

Spatial cross-validation is used to determine a model’s performance for areas that do not share the same spatial characteristics. Spatial cross validation was conducted in a similar fashion to random CV, except for the fact that 17 groups were identified as spatial clusters (Figure 2). The data were split to train/test the dataset based on spatial clusters defined previously using K-mean clustering. We applied GridSearch’s random 10-fold cross validation in the training dataset to tune the hyper-parameters. Then, the defined model was fit in the test dataset to define the prediction values and the evaluation matrix. This process was repeated 17 times. Thus, the resulting evaluation matrix was calculated based on averaging the predicted values from each iteration.

With Blacksburg (VA) being a smaller town with somewhat similar spatial features, splitting the datasets based on specific locations within Blacksburg (e.g., according to census tract ID number) would not be representative. Instead, we chose to use spatial variables (land use and transportation) to segment observational data and define the resulting spatial-split performance. For each set of models (daily average, one second, and hourly average), we clustered the observations based on spatial data using k-means clustering. Unfortunately, no general theoretical solution exists for determining the optimal number of clusters for any given dataset. As such, a straightforward approach is to compare the results of multiple runs with different k classes and select the optimal one based on a predefined criterion. Still, we must exercise caution—not only because k-means clustering can result in smaller error function values, but also because there is a greater risk of overfitting. To overcome this possibility, we first reduced the number of spatial variables to define the most representative variables, and then applied the elbow method (or elbow clustering). We first applied the recursive feature elimination cross-validation RFECV techniques, which identifies several spatial variables that fit a model and removes the weakness features until the most representative feature number is reached. We then applied the “RFECV” provided within the “yellowbrick” library in python using lasso regression. However, prior to this step we first removed all features that had a correlation coefficient above 0.8 (i.e., the most highly correlated features) from the data using a correlation matrix. Then, we defined the best alpha using the “LassoCV” from the same library, after which we used the variables defined by the RFECV to “KElbowVisualizer” from the same library to define the optimal number of clusters. Finally, we applied the “KMeans” module from the scikit-learn [30] library using the most representative variables and the number of defined features.

#### 2.4.3. Spatial–Temporal Cross-Validation

To cluster the data, we used the spatial clusters defined previously and the number of runs for each hour of the day. Specifically, for each hour of day we conducted at least five runs for each route. For each spatial cluster, the final run of each hour for each route was eliminated. Then, the data from the same spatial and temporal cluster was removed from the training data set during the model training process. We applied the same process used for spatial clustering; this process was repeated 17 times.

## 3. Results

This section presents the observed trends from the time-term models from the refined one second measurements model (disaggregated data), the one-hour term model (temporally aggregated 1 h), and the long-term aggregated model (12 h aggregated model). In addition, three types of validation approaches were used to investigate model overestimation by comparing the oriented validation approach with random cross-validation models. Mobile measurement concentrations were averaged, which were determined to be 10,293 pt/cm^3^ for particle number PN and 1.08 μg/m^3^ for BC (Table 2). 

### 3.1. Random Cross Validation Result

Random CV was applied to (a) assess any performance differences between short-term and long-term models; and (b) quantify the overfitting by comparing it with spatial and spatial–temporal CV data. The one second and hourly average models were constructed using two sets of different input variables.

The daily average models (12 h average) models demonstrated good performance across the three-time average models (Table S1). Two types of the hourly average and one second models were constructed based on the different input variables. The goal for the one second dataset (1 s) was not only to define the model in high temporal resolution, but also to show the ability of ML to capture all patterns without the need for data preprocess that are usually applied in traditional statistical models. The average of the MAE (RMSE) of the best one second models was 0.61 μg/m^3^ (1.77 μg/m^3^) for BC and 4991.09 pt/cm^3^ (30,067.64 pt/cm^3^) for PN (Tables S4 and S5). The one second and hourly average models constructed for the land use (Lu) dataset evidenced the worst performance (Table S4). The performance of the one second data model improved from MAE (RMSE) 0.78 (2.35) to 0.44 (1.18) for BC and from 6590.91 (31,280.53) to 3391.27 (28,854.74) for PN when the temporal variables were added (Table S5). The average of the MAE (RMSE) across all types of the best hourly average models was 0.26 μg/m^3^ (0.38 μg/m^3^) for BC, and 1451.17 pt/cm^3^ (2325.96 pt/cm^3^) for PN (Tables S2 and S3). Similar to the one second models, the hourly average models that include both temporal and spatial variables were found to be the most accurate. However, the performance of the hourly average data model was found to improve from MAE (RMSE) 0.3 (0.42) to 0.23 (0.34) for BC and from 1698.33 (2613) to 1204 (2038.31) for PN when the temporal variables were added. This finding indicates that constructing this model using unrefined disaggregated measurements with only spatial land use data should not be considered to be representative of estimating the pollutant concentration. This finding is in line with previous research reinforcing the importance of the temporal variable (weather) in building the PN prediction model [31].

### 3.2. Comparison among Long-Term and Short-Term Models

To investigate the effectiveness and efficiency of ML in detecting pollutant concentration using high resolution data, and to assess its ability to use raw measurement data without data reprocessing, we varied averaging times of the mobile measurements. Therefore, several time-averaging models were implemented to compare the performance between the 1 s measurement models and the longer duration averaging models. For comparison consistency, the conclusions discussed in this section emerged from comparing the two model types. The first comparison pertains to the models that were developed from the same input variables, while the second comparison is based on long-term models versus short-term models that were built using both spatial and temporal variables.

#### 3.2.1. Long-Term vs. Short-Term Spatial Data Models

This section describes the methodology used to achieve comparative consistency in evaluating the models. For the daily average model, averaging the temporal data across 12 h helped to eliminate any variations caused by weather factors. On both short-term models (i.e., hourly average and one second models), the model based on land use as an input data was identified as the poorest-performing model. However, in order to thoroughly explore the differences between the time-averaging models, it is essential to compare models based on the same input variables. As shown in Table 3, the daily average model clearly performed better in comparison to the hourly average model, followed by the one second model. This finding is consistent with previous research [27] indicating that the model fit for the hourly average models (10-fold CV R2: 0.4 for PN and 0.27 for BC) was worse than the daily average models (PN: 0.695; BC: 0.57) for both pollutants. It should be noted that the error differences between the daily average model and hourly average model were not significant. In contrast, there were notable differences between the hourly average model and the one second model. This trend was not surprising since both the daily average and hourly average models underwent time series preprocessing or smoothing in order to eliminate any random variance that tends to be present in data collected over time. It should also be noted that sample size and a lack of spatiotemporal predictor variables may have contributed to differences in model fit between the time-average models.

#### 3.2.2. Long-Term vs. Short-Term Models Based on All Input Variables

Comparing the long-term model with the short-term model that incorporated all input variables was undertaken to determine the advantages of developing one or more models based on the best representative variables—even in the absence of data preprocessing that should be used in traditional statistical analyses. Similar to the findings detailed in Section 3.2.1, the daily average model still demonstrated the best model performance, followed by the hourly average model, and then the one second model (Table 3). However, differences between the long-term and short-term model decreased when we added the temporal variables to the short-term model.

**Figure 3:**
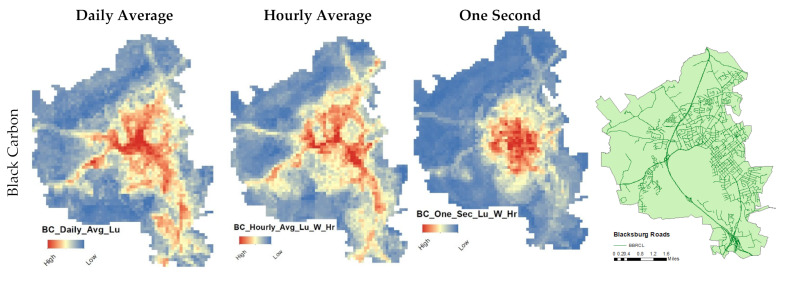

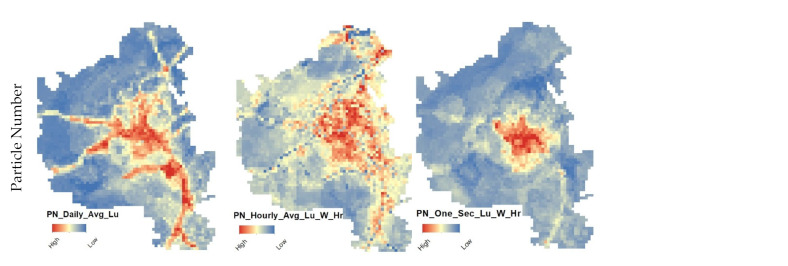
Models estimated standardized concentrations from the three time-averaging models for each pollutant.

**Figure 4:**
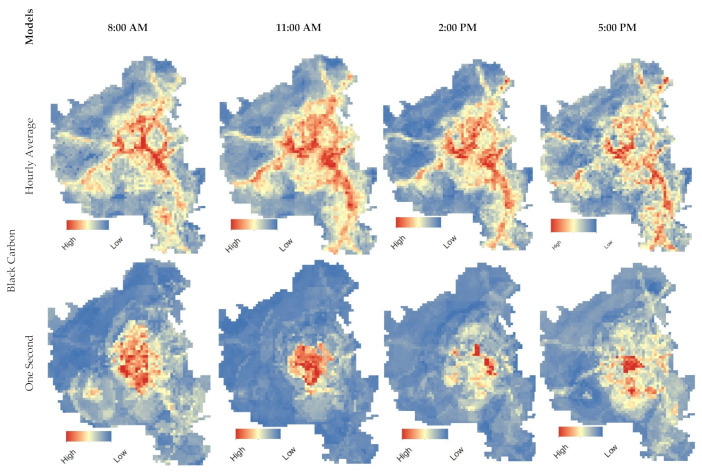

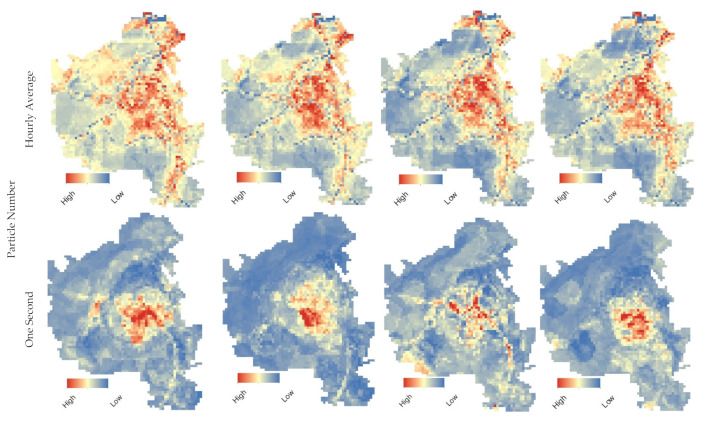
Model estimated standardized concentrations from the hourly average and one second models for select hours of day.

To illustrate spatial prediction from the short-term models, concentration estimates were mapped for selected hours on a 100 m × 100 m grid (Figure 3 and Figure 4). The red areas of the figures show that elevated concentrations of both BC and PN could be found in crowded residential and industrial districts, as well as at road interchanges and along larger interstates.

As shown in Figure 3, all three time-averaging land used data models (daily average, hourly average, and one second) produced models with similar spatial patterns. In contrast, the disaggregated models were unable to detect the street and road emissions for both pollutants; we attribute this finding to noise in the dataset, which was removed when the data were aggregated for daily and hourly averaged models. However, the disaggregated model (one second) was able to detect hotspot areas.

Figure 4 illustrates how pollutant concentration may be higher at certain times of the day than at others. Note that the hourly average models were better at capturing the pollutant concentration distribution in the targeted areas compared to the one second models. This figure also shows that the model was able to detect increases in pollutant concentration at specific times. Specifically, one can see that PN concentrations could be detected at higher levels in comparison to the distribution of BC across the study area.

### 3.3. Machine Learning vs. Statistical Model

The primary distinction between non-parametric ML and parametric statistics is its intended use. Machine learning models use input data to make accurate predictions based on that data. In contrast, statistical models are mathematical equations designed to develop inferences about the relationships between variables. Previous research have employed a 10-fold random CV R^2^ and estimated the performance of stepwise regression models using both daily average and hourly average models [27]. The main goal of this comparative study was to determine the ability of ML to build more accurate models without relying on rules-based programming. To maintain consistency in this study, the comparison of the hourly average model was based on the Hourly_average_Lu_W_Hr models, which featured the same input variables used to build the stepwise regression model.

Overall, it is important to note that the ML models performed better than the stepwise regression across all models (Table 4), which supports the potential utility of ML models. Performance improvements varied between BC and PN data, as well as whether short-term or long-term models were used for each pollutant. The stepwise 10-fold random CV R^2^ of the Daily_average_Lu model was found to be 0.57 for the BC model, and 0.7 for the PN model. Thus, the use of ML models improved the 10-fold CV R^2^ results to 25.8% and 11.4% for BC and PN, respectively.

The stepwise regression hourly average model fit better for PN concentration (10-fold CV R^2^ is 0.42) in comparison to BC concentration (10-fold CV R^2^ is 0.27), but worse when compared to the daily average model results for both pollutants (BC: 0.57; PN: 0.7). Moreover, the ML model was found to determine BC performance with greater precision in comparison to analogous findings for PN; specifically, ML increased the model 10-fold CV R^2^ to twice for the BC and 30% for the PN, which ended up with the same 10-fold CV R^2^ results (0.54 for both BC and PN). In terms of additional BC-related findings, the ML model for the hourly average model was found to be as accurate as the stepwise regression of the daily average model. This finding demonstrates the ability of ML to produce more accurate short-term models that are equivalent with traditional long-term models. Moreover, when comparing the two models, ML was shown to be more effective in developing the BC prediction model in comparison to the PN prediction model.

### 3.4. Spatial and Spatial–Temporal Cross Validation

Three types of cross-validation approaches were applied to show the ability of ML to forecast pollution levels in areas not previously assessed. These types of CV approaches could help researchers to define the accurate CV strategies and validate model reliability. The performance of ML using different types of cross validation strategies will, of course, vary. For instance, the relationship between time of observation and spatial–temporal boundaries can make it difficult to define an accurate validation approach, thereby jeopardizing the reliability of performance evaluations [23]. This factor motivated this research to use more sophisticated validation approaches that would help identify reliable performance patterns in ML, with the goal of predicting concentration levels more accurately. Therefore, this section will discuss the spatial and spatial–temporal cross validation model result.

Spatial cross-validation was used to assess the performance of the prediction model in unseen areas by eliminating locational information in the model training process. Furthermore, spatial–temporal CV was applied to assess the ability of ML to build a forecasting system for pollutant concentration in an unseen location and time. Since not all hourly data were collected on the same day, holding out the last run of each hour of the day will force the data to be balanced. This particular approach was applied only for one second models. The spatial–temporal CV was not applied in the hourly average models because those data points were temporally one-hour aggregated and spatially 100 m aggregated; as such, a forced balancing cannot be applied to this type of aggregation data.

The performance of the daily average model was found to deteriorate from random CV to spatial CV. The MAE spatial CV (RMSE spatial CV) results for the best-performing BC machine learning model were found to be 0.2 μg/m^3^ (0.27 μg/m^3^) (Table S6). The MAE spatial CV (RMSE spatial CV) results for the best PN machine learning models were found to be 886.91 pt/cm^3^ (1338.33 pt/cm^3^) (Table 5). Moreover, the hourly average models’ performance was also found to deteriorate when spatial CV applied. Overall, the average MAE spatial CV (RMSE spatial CV) results across the two types of hourly average best models were found to be 0.35 μg/m^3^ (0.58 μg/m^3^) for BC, and 1932.37 pt/cm^3^ (3113.36 pt/cm^3^) for PN (Table 5, Tables S7 and S8). Similar to the daily average and hourly average deterioration from random CV to spatial CV, the performance of the one second model deteriorated when spatial cross validation was applied. Results for the one second models average MAE spatial CV (RMSE spatial CV) were found to be 0.82 μg/m^3^ (2.49 μg/m^3^) for black carbon, and 6221.24 pt/cm^3^ (30,148.26 pt/cm^3^) for particle number (Tables S9 and S10). As noted previously, spatial–temporal cross validation was applied to the one second data. The average of the MAE spatial–temporal CV (RMSE spatial–temporal CV) data across the two types of one second best models resulted in the following findings: 0.69 μg/m^3^ (1.34 μg/m^3^) for BC and 9518.14 pt/cm^3^ (34,651.64 pt/cm^3^) for PN (Table 5, Tables S11 and S12).

To reiterate, three types of cross-validation approaches were applied to our PN models. The resulting significant increase in error data between the random CV and spatial–temporal CV approaches indicates spatial–temporal overfitting. We attribute this outcome to the strategy of removing data from the same spatial–temporal cluster in the training set, which caused the results to be less robust compared to a spatial CV wherein more data were available for training the model. These trends were found to deviate in the case of the black carbon models; specifically, the spatial CV models performed worse in comparison to the random hold-out models. In contrast, the spatial–temporal CV models performed better in comparison to the spatial CV models, which represents an unexpected finding. This outcome indicates that the spatial CV test dataset featured noise, which was reduced when we increased the limitation of the hold-out set; thus, with a reduction in test set size, the noise decreased. This finding can be attributed to the inherent noise associated with the microaethalometer that was used to collect black carbon concentration data.

## 4. Discussion

We developed three types of time-averaging models which were fed temporal information based on data obtained from a comprehensive mobile monitoring approach along two bike routes in rural college town in Blacksburg. We applied three types of cross validation models (randomly, spatially, and spatially temporally) to assess the model overestimation. The main goal of this study was to test different data reprocessing approaches using mobile-monitoring data with the goal of assessing the ability of ML to improve prediction accuracy using high temporal resolution monitoring data (1 s). Our results indicated that the aggregation data models perform better than the disaggregated data models. Among the three types of time-averaging models, the daily average model evidenced the least error across the two types of cross validation the MAE for BC and PN were 0.11 μg/m^3^ and 515.2 pt/cm^3^ for random CV and 0.2 μg/m^3^ and 886 pt/cm^3^ for spatial cross validation, respectively. The average random CV MAE of the hourly average models dropped to 57.2% for BC and 70.9% for PN when compared to the average MAE of the one second models, indicating that the hourly average aggregation data fit better than the raw data. This finding can be attributed to the fact that the data were spatially and temporally smoothed and the noise was removed. Indeed, smoothing techniques are extremely useful in ML because conditional expectations can be viewed as trends of unknown shapes that must be predicted when uncertainty parameters are present.

Multiple studies have used ML to produce accurate predictions with high spatial–temporal resolution [32]. Nonetheless, while mobile monitoring can produce data with high spatial resolution, its ability to provide similarly rich temporal resolution information for the same monitoring location is limited, especially over shorter monitoring periods. Therefore, researchers must continue to explore the performance of ML in the presence of different data pre-processing approaches to develop accurate models using high temporal resolution mobile monitoring data (one-second measurement data). This study compared the performance of the ML models of the refined versus the spatially and temporally smoothed input data. The results demonstrated the consistency of the two time-average models in detecting the spatial distribution of PN and BC. However, the one second model was not able to capture pollutant concentrations with the same equivalency as the daily average and the hourly average models. Nevertheless, the one second models were able to detect the hotspot area, indicating the ability of ML to handle noisy, unprocessed mobile monitoring data. It must be noted that this study used secondary data captured during the summer and fall within a smaller, more rural town with less urban diversity. Thus, differences in spatial and temporal factors were not found to be significant. A follow-on study is recommended to expand these findings by conducting a similar study in a more urban locale and for a longer period. When compared to the distribution of BC over the study area, PN concentrations were found to be at greater levels. This finding supports previous research findings [17]. According to that research, geographical coefficients imply that PN concentrations are more influenced by background concentrations than BC.

Additionally, comparing ML models and traditional statistical models (stepwise regression) was undertaken to assess the ability of using ML to model accuracy. Our findings indicate that ML demonstrated higher predictive capacity with respect to stepwise regression. Additionally, models used to predict black carbon levels were more accurate than those applied to predicting particle numbers.

Finally, many of the land-use studies have applied random validation to test the reliability and uncertainty of the model. However, it has been shown that applying another strategy—namely cross-validation—could help to overcome spatiotemporal autocorrelation [33]. Therefore, three types of cross-validation approaches were applied to show the ability of ML to forecast pollution levels in an area not previously assessed. The different cross-validation approaches could help to address problems of overestimation that probably occurred in the random cross-validation model. Between the random, spatial, and spatiotemporal types of cross-validation approaches that were applied to the BC (PN) hourly average models, the average MAE values for both the land use and all-variables models increased from 0.27 μg/m^3^ (1451.2 pt/cm^3^) using the random CV approach, to 0.356 μg/m^3^ (1932.6 pt/cm^3^) using the spatial CV approach. Furthermore, across the three types of cross-validation approaches that were applied to BC (PN), for one-second disaggregated models, the average MAE values for both the land use and all-variables model increased from 0.61 μg/m^3^ (4991.1 pt/cm^3^) using the random CV approach, to 0.825 μg/m^3^ (6221.24 pt/cm^3^) using the spatial CV approach, and to 0.69 μg/m^3^ (9518.15 pt/cm^3^) using the spatial–temporal CV approach from random CV. In general, prediction mean absolute errors increased from random to spatial cross-validation. This finding is in line with previous research [20,21]; this difference indicates that the model became less capable of predicting beyond the training data’s immediate location compared to what might have been expected using random CV. Previously, researchers used machine learning to construct prediction models with spatial and temporal structure, introducing spatiotemporal autocorrelation [24]. Our result shows that the model’s mean absolute error rate increased from using spatial cross-validation to using spatial–temporal cross-validation. This significant increase in error data between the random CV and spatial–temporal CV approaches indicates spatial–temporal overfitting. To reiterate, however, these data points were collected during a period when there would typically be less traffic. Therefore, the findings discussed herein may not be representative of more annualized pollutant levels. Accordingly, a similarly designed study should be undertaken during the periods of higher traffic density to detect pollutant concentrations. Moreover, more effort should be taken to discuss model overfitting solutions.

## 5. Conclusions

Three time-interval models (two short-term models and one long-term model) were used to estimate the concentrations of the pollutants PN and BC in Blacksburg (VA) utilizing mobile-monitoring data, involving spatial and temporal variables. The main goal of this study was to test different data reprocessing approaches for the mobile monitoring data to assess the ability of ML to improve prediction accuracy using high temporal resolution monitoring data (1 s). Furthermore, three type of cross validation models were applied to detect the ability of the model to predict the concentration beyond the study area and time.

We altered the averaging times of the mobile measurements to evaluate the effectiveness and efficiency of machine learning in detecting pollutant concentrations using high-resolution data and assessing its capacity to use raw measurement data without data reprocessing. To compare the performance of the 1 s measurement models and the longer length averaging models, three time-averaging models were created. The purpose of comparing the long-term and short-term models was to establish the benefits of building a machine learning model even in the lack of data preprocessing, which is typically utilized in traditional statistical analyses. The results showed that the two time-average models were equally good at detecting the geographical distribution of PN and BC. The one second model, on the other hand, was unable to capture pollutant concentrations as well as the daily average and hourly average models. Despite this, the one second models were able to pinpoint the hotspot area, demonstrating that machine learning can handle noisy, unprocessed mobile monitoring data.

The significant increase in error data between the random CV and spatial–temporal CV approaches indicates spatial–temporal overfitting. We ascribe this result to the training set’s strategy of deleting data from the same spatial–temporal cluster. When compared to a spatial CV, where more data are available for training the model, the findings tend to be less reliable.

In conclusion, holding out spatial and temporal data may be helpful in understanding the correlation between CV bias and error underestimation; it must be noted that the systematic validation approach may be very sensitive to the test size. However, predictions may be enhanced by carefully considering the spatial–temporal dimension in training. The results detailed herein support the importance of defining validation strategies; indeed, the results obtained from this investigation confirm that the type of validation technique(s) plays a critical role in evaluating a model’s reliability.

## Figures and Tables

**Table 1:** Summary of variables used and CV applies to develop the daily average, one second, and hourly average empirical models.

Pullutant	LUR Models	Input Variables	Temporal Resolution	Number of Observations	Input Variables			Cross_Validation		
					Land Use, Transportation, Natural Envirnoment	Weather	Hour of the Day	Random_Holdout	Spatial_Holdout	Spatial_Temporal_Holdout
BC: µg/m^3^, PN: particles/cm	Daily Average	Lu	12 h	423	☑			☑	☑	
	Hourly Average	Lu	1 h	5076	☑			☑	☑	
		Lu_W_Hr			☑	☑	☑	☑	☑	
	One second	Lu	1 s	BC: 319,490 -PN: 354,717	☑			☑	☑	☑
		Lu_W_Hr			☑	☑	☑	☑	☑	☑

**Table 2:** Summary of Pollutant Descriptive Statistics.

Pollutant	Models	Count	Min	Q1	Q3	Max	Mean	Median	S.D.
Black Carbon (μg/m^3^)	Daily average	423	0.17	0.47	0.81	1.63	0.67	0.62	0.27
	Hourly average	5074	−0.64	0.41	0.95	16.76	0.74	0.63	0.59
	One second	319,489	−6.56	0.34	1.21	108.01	1.08	0.69	2.49
Particle Number (pt/cm^3^)	Daily average	423	4305	5430	6925	14,382	6388	5924	1491
	Hourly average	5074	1104	4996	7679	41,281	6834	6044	3205
	One second	354,715	4	3420	11338	4,447,494	10,293	5950	30,139

**Table 3:** Summary of model performance for each pollutant and model type in the random CV.

Models	Input Variables	BC		PN	
		Random CV		Random CV	
		MAE (μg/m^3^)	RMSE (μg/m^3^)	MAE (pt/cm^3^)	RMSE (pt/cm^3^)
Daily average	Lu	0.11	0.15	515.21	765.48
Hourly average	Lu	0.30	0.42	1698.33	2613.62
	Lu_W_Hr	0.23	0.34	1204.00	2038.31
One second	Lu	0.78	2.35	6590.91	31,280.53
	Lu_W_Hr	0.44	1.18	3391.27	28,854.74

**Table 4:** Comparison between Machine Learning and Stepwise Regression Performance for Each Pollutant.

Pollutant	Models	Stepwise R^2^	ML R^2^
BC: µg/m^3^	Daily_average (Lu)	0.58	0.734
	Hourly_average (Lu + W + Hr)	0.27	0.54
PN: particles/cm^3^	Daily_average (Lu)	0.7	0.78
	Hourly_average (Lu + W + Hr)	0.42	0.54

**Table 5:** Summary of the spatial and spatial-temporal of cross validation for BC and PN.

Models	Input Variables	BC (μg/m^3^)				PN (pt/cm^3^)			
		Spatial CV		Spatial–Temporal		Spatial CV		Spatial–Temporal	
		MAE	RMSE	MAE	RMSE	MAE	RMSE	MAE	RMSE
Daily average	Lu	0.20	0.27			886.91	1338.33		
Hourly average	Lu	0.36	0.59			2002.01	3204.07		
	Lu W Hr	0.35	0.58			1862.72	3022.66		
One second	Lu	0.84	2.50	0.71	1.35	6831.35	30,366.65	10,145.75	34,931.82
	Lu W Hr	0.81	2.48	0.67	1.33	5611.13	29,929.87	8890.51	34,371.46

## Supplementary Materials

The following are available online at https://www.mdpi.com/article/10.3390/ijerph191610098/s1, Table S1. Summary of Random CV Model Performance for Daily_average Models; Table S2. Summary of Random CV Model Performance for Hourly_average_Land_Use Models; Table S3. Summary of Random CV Model Performance for Hourly_average_Land_Use_Weather_Hour_of_Day Models; Table S4. Summary of Random CV Model Performance for One_second_Land_Use Models; Table S5. Summary of Random CV Model Performance for One_second_Land_Use_Weather_Hour_of_Day Models; Table S6. Summary of Spatial CV Model Performance for Daily_average Models; Table S7. Summary of Spatial CV Model Performance for Hourly_average_Land_Use_Weather_Hour_of_ Day Models; Table S8. Summary of Spatial CV Model Performance for Hourly_average_Land_Use Models; Table S9. Summary of Spatial CV Model Performance for One_second_Land_Use Models; Table S10. Summary of Spatial CV Model Performance for One_second_Land_Use_Weather_Hour_of_Day Models; Table S11. Summary of Spatial-temporal CV Model Performance for One_second_Land_Use Models; Table S12. Summary of Spatial-temporal CV Model Performance for One_second_Land_Use_Weather_Hour_of_Day Models.
