# Supplementary material for: Assessing and Validating the Ability of Machine Learning to Handle Unrefined Particle Air Pollution Mobile Monitoring Data Randomly, Spatially, and Spatiotemporally
Source: Int J Environ Res Public Health. 2022 Aug 16;19(16):10098. doi: 10.3390/ijerph191610098 (PMC9408314; doi:10.3390/ijerph191610098)
Supplement: Supplementary file 1 [file ijerph-19-10098-s001.zip › ijerph-1672372-supplementary.pdf]

Table S1. Summary of Random CV Model Performance for Daily_average Models.

BC (μg/m³)			PN (pt /cm³)		
**Models**	**MAE**	**RMSE**	**Models**	**MAE**	**RMSE**
Decision Tree	0.15	0.19	Decision Tree	609.63	1085.84
Extra Tree	0.14	0.19	Extra Tree	591.31	946.74
K Neighbors	0.13	0.17	K Neighbors	505.62	865.64
SVR	0.15	0.18	SVR	589.35	841.91
Ridge	0.13	0.18	Ridge	515.21	765.48
Elastic Net	0.13	0.17	Elastic Net	568.20	978.91
Lasso	0.17	0.21	Lasso	634.36	1147.87
Random Forest	0.12	0.16	Random Forest	515.77	1002.66
Gradient Boosting	0.11	0.15	Gradient Boosting	461.64	930.17

Table S2. Summary of Random CV Model Performance for Hourly_average_Land_Use Models.

BC (μg/m³) (Hourly_average_Lu)			PN (pt /cm³) (Hourly_average_Lu)		
**Models**	**MAE**	**RMSE**	**Models**	MAE	RMSE
Decision Tree	0.31	0.44	Decision Tree	1770.99	2681.53
Extra Tree	0.31	0.44	Extra Tree	1787.09	2665.32
K Neighbors	0.30	0.42	K Neighbors	1760.95	2613.73
Ridge	0.30	0.42	Ridge	1795.71	2668.26
Elastic Net	0.30	0.42	Elastic Net	1721.93	2635.43
Lasso	0.31	0.45	Lasso	1748.71	2733.27
Random Forest	0.30	0.42	Random Forest	1698.33	2613.62
Gradient Boosting	0.30	0.42	Gradient Boosting	1700.97	2623.10

Table S3. Summary of Random CV Model Performance for Hourly_average_Land_Use_Weather_Hour_of_Day Models.

BC (μg/m³) (Hourly_average_Lu_W_Hr)			PN (pt /cm³) (Hourly_average_Lu_W_Hr)		
**Models**	**MAE**	**RMSE**	**Models**	**MAE**	**RMSE**
Decision Tree	0.31	0.43	Decision Tree	1712.87	2579.69
Extra Tree	0.30	0.42	Extra Tree	1588.06	2451.72
K Neighbors	0.26	0.39	K Neighbors	1543.28	2440.11
SVR	0.26	0.37	SVR	1385.87	2314.83
Ridge	0.28	0.40	Ridge	1514.54	2393.25
Elastic Net	0.27	0.40	Elastic Net	1468.70	2384.65
Lasso	0.29	0.43	Lasso	1555.62	2532.63
Random Forest	0.28	0.41	Random Forest	1588.10	2449.36
Gradient Boosting	0.23	0.34	Gradient Boosting	1216.85	2058.4

Table S4. Summary of Random CV Model Performance for One_second_Land_Use Models.

BC (μg/m³) (One_second_Lu)			PN (pt /cm³) (One_second_Lu)		
**Models**	**MAE**	**RMSE**	**Models**	MAE	RMSE
Decision Tree	0.79	2.35	Decision Tree	6598.13	31286.34
Extra Tree	0.79	2.35	Extra Tree	6602.49	31293.71
K Neighbors	0.84	2.37	K Neighbors	6720.78	31288.53
Ridge	0.79	2.36	Ridge	6612.34	31301.13
Elastic Net	0.79	2.36	Elastic Net	6621.46	31316.65
Lasso	0.80	2.40	Lasso	6641.62	31350.75
Random Forest	0.78	2.35	Random Forest	6590.91	31280.53
Gradient Boosting	0.78	2.35	Gradient Boosting	6592.56	31282.66

Table S5. Summary of Random CV Model Performance for One_second_Land_Use_Weather_Hour_of_Day Models.

BC (μg/m³)	(One_second_Lu_W_Hr)		PN (pt /cm³)	(One_second_Lu_W_Hr)	
**Models**	**MAE**	**RMSE**	**Models**	**MAE**	**RMSE**
Decision Tree	0.67	1.76	Decision Tree	5301.30	30673.95
Extra Tree	0.77	2.23	Extra Tree	6185.16	31109.89
Ridge	0.74	2.33	Ridge	5577.72	31101.78
Elastic Net	0.74	2.34	Elastic Net	5605.19	31136.33
Lasso	0.77	2.39	Lasso	5662.80	31197.51
Random Forest	0.64	1.87	Random Forest	4708.96	30487.32
Gradient Boosting	0.44	1.18	Gradient Boosting	3391.27	28854.74

Table S6. Summary of Spatial CV Model Performance for Daily_average Models.

BC (μg/m³)			PN (pt /cm³)		
**Models**	**MAE**	**RMSE**	**Models**	**MAE**	**RMSE**
Decision Tree	0.24	0.30	Decision Tree	1132.43	1593.33
Extra Tree	0.24	0.30	Extra Tree	1152.54	1696.72
K Neighbors	0.20	0.27	K Neighbors	886.91	1338.33
SVR	0.21	0.28	SVR	1041.54	1415.10
Ridge	0.26	0.34	Ridge	1224.92	1615.21
Elastic Net	0.21	0.27	Elastic Net	985.90	1391.02
Lasso	0.21	0.27	Lasso	999.08	1390.64
Random Forest	0.21	0.27	Random Forest	976.83	1384.41
Gradient Boosting	0.21	0.27	Gradient Boosting	1019.84	1424.12

Table S7. Summary of Spatial CV Model Performance for Hourly_average_Land_Use_Weather_Hour_of_ Day Models.

BC (μg/m³) (Hourly_average_Lu_W_Hr)			PN (pt /cm³) (Hourly_average_Lu_W_Hr)		
**Models**	**MAE**	**RMSE**	**Models**	**MAE**	**RMSE**
Decision Tree	0.45	0.66	Decision Tree	2374.10	3586.52
Extra Tree	0.39	0.63	Extra Tree	2227.96	3520.13
K Neighbors	0.36	0.59	K Neighbors	1903.67	3057.31
SVR	0.52	0.80	SVR	2729.56	6634.06
Ridge	0.57	0.86	Ridge	3488.46	5586.09
Elastic Net	0.36	0.59	Elastic Net	1951.56	3022.73
Lasso	0.35	0.59	Lasso	1862.72	3022.66
Random Forest	0.35	0.58	Random Forest	1949.06	3148.33
Gradient Boosting	0.35	0.58	Gradient Boosting	1917.43	3061.03

Table S8. Summary of Spatial CV Model Performance for Hourly_average_Land_Use Models.

BC (μg/m³) (Hourly_average_Lu)			PN (pt /cm³) (Hourly_average_Lu)		
**Models**	**MAE**	**RMSE**	**Models**	**MAE**	**RMSE**
Decision Tree	0.41	0.63	Decision Tree	2492.20	3701.17
Extra Tree	0.42	0.65	Extra Tree	2462.84	3742.51
K Neighbors	0.38	0.60	K Neighbors	2016.49	3192.74
Ridge	0.58	0.87	Ridge	3706.32	5660.79
Elastic Net	0.41	0.63	Elastic Net	2123.79	3272.51
Lasso	0.36	0.59	Lasso	2026.28	3221.27
Random Forest	0.36	0.59	Random Forest	2002.01	3204.07
Gradient Boosting	0.36	0.59	Gradient Boosting	2049.63	3256.10

Table S9. Summary of Spatial CV Model Performance for One_second_Land_Use Models.

BC (μg/m³) (One_second_Lu)			PN (pt /cm³) (One_second_Lu)		
**Models**	**MAE**	**RMSE**	**Models**	**MAE**	**RMSE**
Decision Tree	0.91	2.53	Decision Tree	6904.57	30397.43
Extra Tree	0.88	2.51	Extra Tree	6964.44	30377.66
Ridge	1.49	2.97	Ridge	8170.86	32009.87
Elastic Net	0.84	2.49	Elastic Net	6910.54	30380.15
Lasso	0.84	2.50	Lasso	6842.79	30372.20
Random Forest	0.84	2.49	Random Forest	6831.35	30366.65

Table S10. Summary of Spatial CV Model Performance for One_second_Land_Use_Weather_Hour_of_Day Models.

BC (μg/m³) (One_second_Lu_W_Hr)			PN (pt /cm³) (One_second_Lu_W_Hr)		
**Models**	**MAE**	**RMSE**	**Models**	**MAE**	**RMSE**
Decision Tree	0.84	2.51	Decision Tree	6567.61	30284.32
Extra Tree	0.88	2.53	Extra Tree	6586.31	30261.41
Ridge	1.44	2.93	Ridge	7921.65	32069.72
Elastic Net	0.81	2.48	Elastic Net	5697.73	29974.11
Lasso	0.81	2.48	Lasso	5766.65	30027.63
Random Forest	0.82	2.47	Random Forest	5611.13	29929.87

Table S11. Summary of Spatial-temporal CV Model Performance for One_second_Land_Use Models.

BC (μg/m³) (One_second_Lu)			PN (pt /cm³) (One_second_Lu)		
**Models**	**MAE**	**RMSE**	**Models**	**MAE**	**RMSE**
Decision Tree	0.88	1.49	Decision Tree	10295.46	34880.21
Extra Tree	1.04	2.04	Extra Tree	10324.67	35018.02
Ridge	1.38	2.10	Ridge	11115.64	35595.92
Elastic Net	0.71	1.34	Elastic Net	10199.22	34981.08
Lasso	0.71	1.35	Lasso	10148.96	34967.34
Random Forest	0.72	1.34	Random Forest	10145.75	34931.82

Table S12. Summary of Spatial-temporal CV Model Performance for One_second_Land_Use_Weather_Hour_of_Day Models.

BC (μg/m³) (One_second_Lu_W_Hr)			PN (pt /cm³) (One_second_Lu_W_Hr)		
**Models**	**MAE**	**RMSE**	**Models**	**MAE**	**RMSE**
Decision Tree	0.82	1.78	Decision Tree	10361.44	35042.39
Extra Tree	0.81	2.30	Extra Tree	10020.68	34890.73
Ridge	1.31	2.03	Ridge	10999.58	35655.79
Elastic Net	0.69	1.34	Elastic Net	8890.51	34371.46
Lasso	0.67	1.33	Lasso	8916.41	34526.65
Random Forest	0.72	1.34	Random Forest	9867.21	34867.71
